# Propolis and Caffeic Acid Phenethyl Ester Attenuate Oxidative and Nitrosative Stress in Rat Kidney Tissue After Cranial Irradiation

**DOI:** 10.3390/life16071180

**Published:** 2026-07-16

**Authors:** Aziz Bulut, Oztekin Cikman, Seyithan Taysi

**Affiliations:** 1 Department of General Surgery, Medical School, Gaziantep University, 27310 Gaziantep, Turkey; azizbulut@gantep.edu.tr; 2 Department of General Surgery, Medical School, Yuzuncu Yil University, 65080 Van, Turkey; droztekin65@gmail.com; 3 Department of Medical Biochemistry, Medical School, Gaziantep University, 27310 Gaziantep, Turkey; 4 Phytotherapy and Medicinal-Aromatic Plants Application and Research Center, Gaziantep University, 27310 Gaziantep, Turkey

**Keywords:** caffeic acid phenethyl ester, propolis, antioxidant enzymes, irradiation, oxidative stress, nitrosative stress, bystander effect

## Abstract

Radiation-induced bystander effect (RIBE) contributes to oxidative and nitrosative stress in non-irradiated tissues following ionizing radiation (IR). This study investigated the protective effects of propolis and caffeic acid phenethyl ester (CAPE) against RIBE-mediated renal injury after total cranial irradiation in rats. Forty-eight male Sprague–Dawley rats were assigned to six groups: IR, IR + propolis, IR + CAPE, propolis control, CAPE control, and sham. A single 5 Gy cranial gamma irradiation was administered. Propolis (80 mg/kg/day, orally) and CAPE (10 μmol/kg/day, intraperitoneally) were given for 10 days. Renal antioxidant (SOD, GSH-Px, GST), oxidative (XO, MDA), and nitrosative (NOS, NO) parameters were evaluated. Multivariate analyses, including principal component analysis (PCA) and partial least squares-discriminant analysis (PLS-DA), were performed. IR significantly decreased SOD activity and increased XO, MDA, NOS, and NO levels. Both propolis and CAPE effectively reversed these alterations, with propolis showing a more pronounced effect on oxidative stress markers. GSH-Px and GST activities remained unchanged. The findings suggest that propolis and CAPE attenuated oxidative/nitrosative changes in kidney tissue, consistent with a possible bystander-associated systemic response.

## 1. Introduction

Radiotherapy (RT), one of the fundamental approaches in the treatment of solid tumors in modern oncology, induces cell death by generating DNA damage in tumor cells through ionizing radiation. However, the biological effects of radiation are not limited solely to directly targeted cells [1,2,3,4,5]. A substantial portion of radiation-induced cellular damage is associated with the excessive production of reactive oxygen species (ROS) and reactive nitrogen species (RNS), leading to oxidative stress, inflammation, and cellular dysfunction [6,7]. These processes result in molecular-level damage, including DNA strand breaks, lipid peroxidation, and protein oxidation, thereby producing significant biological effects in both tumor tissues and normal tissues [3,6].

In recent years, one of the most notable concepts in radiation biology is the indirect radiation response known as radiation-induced bystander effect (RIBE). This phenomenon is defined as the ability of non-irradiated cells to exhibit biological responses through signals released from neighboring irradiated cells. RIBE is mediated by various factors, including cytokines, growth factors, ROS, nitric oxide, and extracellular vesicles, and is characterized by outcomes such as DNA damage, genomic instability, apoptosis, and inflammation [6]. This process can extend the off-target effects of radiotherapy, thereby contributing to normal tissue toxicity. Therefore, the development of agents capable of modulating not only direct radiation-induced damage but also bystander effects is of considerable importance [6,8]. Therefore, natural antioxidant compounds have emerged as promising agents for mitigating radiation-induced damage. Propolis, a bee-derived product, is a complex natural substance containing numerous bioactive constituents, including flavonoids, phenolic acids, aromatic esters, and terpenes. The antioxidant, anti-inflammatory, antimicrobial, and anticancer properties of propolis have been extensively documented. In particular, its ability to suppress oxidative stress, scavenge free radicals, and modulate inflammatory pathways constitutes the basis of its radioprotective potential [7,9,10,11].

Caffeic acid phenethyl ester (CAPE), one of the principal bioactive constituents of propolis, has attracted considerable attention due to its potent antioxidant and anti-inflammatory properties. The primary mechanisms of action of CAPE include inhibition of the nuclear factor-kappa B (NF-κB) signaling pathway, reduction in ROS production, and modulation of apoptotic signaling pathways [12]. These properties may play a critical role in limiting both direct radiation-induced damage and secondary cellular responses associated with the RIBE [6]. Indeed, experimental studies have demonstrated that CAPE can attenuate bystander effects by suppressing oxidative and inflammatory mediators involved in intercellular signaling [7].

Given the central role of oxidative stress and inflammation in the pathophysiology of RIBE, the capacity of antioxidant compounds such as propolis and CAPE to modulate these processes is of particular significance [2,7,8]. It is well established that signals released from irradiated cells can enhance ROS production in neighboring cells, thereby initiating a cascade of propagating cellular damage. The free radical–scavenging activity of propolis, together with the regulatory effects of CAPE on intracellular signaling pathways, may contribute to limiting this propagation. Furthermore, these compounds have been shown to support cellular antioxidant defense systems, such as superoxide dismutase (SOD), catalase (CAT), and glutathione peroxidase (GSH-Px), and to reduce the production of pro-inflammatory cytokines [9,13].

Radiotherapy-induced cellular damage is mediated not only by the direct effects of ionizing radiation (IR) but also by indirect mechanisms such as the RIBE. These two natural compounds have been reported to exert a broad spectrum of molecular effects in the lungs, liver, and gonads of animals exposed to IR, and IR-related adverse effects may persist long after irradiation [6,8]. However, data regarding the radiomodulatory potential of propolis and CAPE in renal tissue remain limited. Therefore, the present study aimed to investigate whether systemic administration of these two natural compounds exerts a protective effect against radiation-induced oxidative and nitrosative damage in kidney tissue located outside the irradiation field.

## 2. Materials and Methods

All experimental procedures were approved by the Gaziantep University Animal Ethics Committee (Approval No: 2017/2) and performed in accordance with the established ethical standards. The selected doses of propolis and CAPE were determined based on previously published studies [6,14]. This study was designed, conducted, and reported in accordance with the Animal Research: Reporting of In Vivo Experiments (ARRIVE) 2.0 guidelines.

A total of 48 male albino Sprague–Dawley rats (12–16 weeks old, weighing 220 ± 25 g at the time of irradiation) were included in this study. The animals were obtained from and maintained at the Experimental Animal Laboratory of the Faculty of Medicine, Gaziantep University. Before radiation exposure, all rats were acclimatized for at least seven days and housed individually. They were kept under standardized environmental conditions, including a controlled ambient temperature of 22 ± 1 °C and a 12 h light/dark cycle, with free access to standard laboratory chow and water. All experimental procedures were conducted in accordance with established ethical guidelines and approved by the relevant institutional authorities.

The animals were randomly assigned to six experimental groups (n = 8 per group) using a randomized allocation protocol:

IR group: Animals were subjected to a single 5 Gy dose of gamma irradiation targeted to the cranial region. Immediately following irradiation, 1 mL of saline was administered via orogastric gavage.

IR + Propolis group: Rats received a single 5 Gy cranial gamma irradiation. Propolis was administered at a dose of 80 mg/kg/day by orogastric gavage, initiated 1 h prior to irradiation and continued daily for 10 days post-exposure.

IR + CAPE group: Animals were exposed to a single 5 Gy dose of cranial gamma irradiation. CAPE was administered intraperitoneally at 10 μmol/kg/day, beginning 30 min before irradiation and maintained for 10 consecutive days. The compound was prepared in dimethyl sulfoxide (DMSO) at a final concentration of 0.1%.

Propolis control group: Rats received 1 mL/day of saline via orogastric gavage for 10 days, without exposure to irradiation or active compounds.

CAPE control group: Animals were administered daily intraperitoneal injections of DMSO for 10 days, at volumes equivalent to those used in the IR + CAPE group. No irradiation or active treatment was applied.

Sham group: Rats in this group were neither irradiated nor treated with propolis, CAPE, or any vehicle.

### 2.1. Anesthesia and Irradiation Procedure

All animals were anesthetized with ketamine hydrochloride (80 mg/kg; Ilac, Istanbul, Turkey) and xylazine (10 mg/kg; Bayer Turk Kimya San. Ltd. Sti., Istanbul, Turkey) [14], and positioned in the prone position on a dedicated irradiation platform. Cranial irradiation was delivered using a cobalt-60 teletherapy system (Theratron Equinox, MDS Nordion, Kanata, ON, Canada) at a source-to-surface distance of 80 cm, employing a 5 × 5 cm field size and a dose rate of 0.49 Gy/min. Dosimetric calibration was performed in accordance with previously established protocols, with the prescribed dose determined along the central beam axis at a depth of 0.5 cm.

### 2.2. Biochemical Analysis

All animals were sacrificed under deep anesthesia induced by ketamine hydrochloride and xylazine, and kidney tissues were collected 10 days following total cranial irradiation. The harvested kidneys were immediately processed and homogenized in ice-cold isotonic saline (IKANERKE GmbH & Co. KB, D-79219, Staufen, Germany). The homogenates were subsequently centrifuged at 10,000× *g* for 60 min to remove cellular debris. The resulting supernatants were carefully collected and used for the determination of all biochemical parameters. All procedures were performed at 4 °C to preserve enzymatic activity and prevent degradation.

### 2.3. Determination of Biochemical Parameters

The activities of SOD, GSH-Px, glutathione S-transferase (GST), and xanthine oxidase (XO) were determined using previously validated spectrophotometric methods, as described in the literature [15,16,17,18]. Enzyme activities were normalized to protein content and expressed as U/mg protein. Lipid peroxidation and nitrosative stress were assessed by measuring malondialdehyde (MDA), nitric oxide (NO^•^) levels, and nitric oxide synthase (NOS) activity, respectively, using established protocols [19,20,21]. Tissue protein concentrations were also quantified according to standard methods [22]. MDA and NO^•^ levels were expressed as µmol/g wet tissue. All biochemical measurements were performed using a spectrophotometer (BioTek Instruments, Winooski, VT, USA) in accordance with the manufacturer’s instructions.

### 2.4. Statistical Analysis

All data were initially evaluated using descriptive statistical methods and are presented as mean ± standard deviation (SD). The normality of data distribution was assessed using the Shapiro–Wilk test. Since the variables showed normal distribution, comparisons among groups were performed using one-way analysis of variance (ANOVA). When a statistically significant difference was detected, pairwise comparisons were conducted using Tukey’s honestly significant difference (HSD) post hoc test to control for multiple comparisons. Relationships between variables were analyzed using Spearman’s rank correlation coefficient. Correlation analyses were performed as exploratory analyses to evaluate possible relationships among antioxidant, oxidative, and nitrosative stress parameters within each experimental group. Given the relatively small sample size in each group and the multiple correlation tests performed, *p*-values obtained from correlation analyses were adjusted using the Benjamini–Hochberg false discovery rate (FDR) method. FDR-adjusted *p*-values < 0.05 were considered statistically significant. All statistical analyses were carried out using SPSS software (version 22.0; IBM Corp., Armonk, NY, USA), and a *p*-value < 0.05 was considered statistically significant.

Multivariate data analyses were conducted using the MetaboAnalyst platform (version 6.0; McGill University, Montreal, QC, Canada) [23]. Before analysis, the dataset was preprocessed by median normalization, log10 transformation, and auto-scaling (mean-centering followed by division by the standard deviation). Principal component analysis (PCA) was applied to explore the overall variance structure of the dataset, while group differences were assessed using permutational multivariate analysis of variance (PERMANOVA). Variables contributing to group discrimination were identified with partial least squares discriminant analysis (PLS-DA) and corresponding variable importance in projection (VIP) scores. Model robustness and validity were evaluated k-fold cross-validation and permutation testing (n = 1000). In addition, hierarchical cluster analysis (HCA) was performed to identify patterns and similarities among variables.

## 3. Results

### 3.1. Antioxidant Parameters

The evaluation of antioxidant enzyme activities revealed no statistically significant differences in GSH-Px and GST levels across the experimental groups. In contrast, SOD activity demonstrated marked intergroup variability, with the lowest values observed in the IR group and the highest in the IR + propolis group (*p* < 0.001). Although not reaching statistical significance in all comparisons, SOD activity in the IR group tended to be reduced relative to the control groups. Notably, SOD activity was significantly decreased in the IR group compared with both the IR + CAPE and IR + propolis groups (*p* < 0.001), suggesting that both propolis and CAPE exert a protective effect against ionizing radiation–induced impairment of antioxidant defenses (Table 1).

### 3.2. Oxidative Stress Parameters

**Figure 1:**
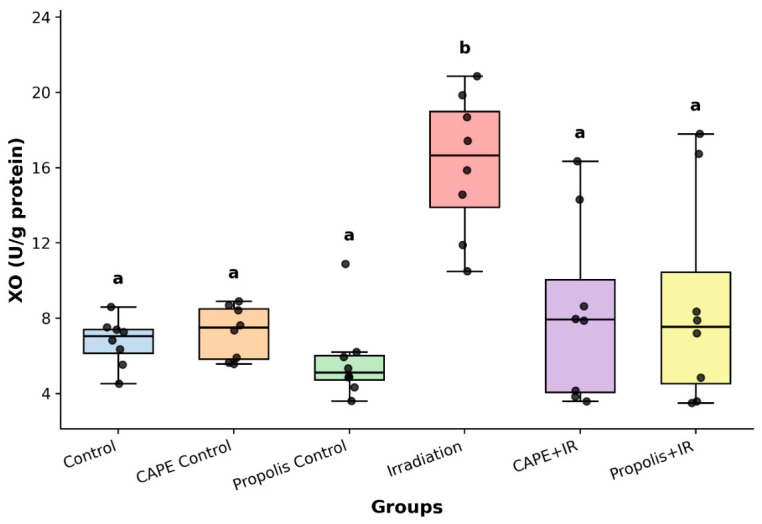
Mean ± SD of XO activity measured in the kidney of the rats (In box plots, if the letters on the boxes representing the groups are the same, there is no difference; if the letters are not the same, there is a difference between them). XO activity was significantly increased in the Irradiation group compared with all other groups. CAPE and propolis treatment significantly reduced irradiation-induced XO activity, bringing the values closer to the control groups. Groups sharing the same letter were not significantly different.

**Figure 2:**
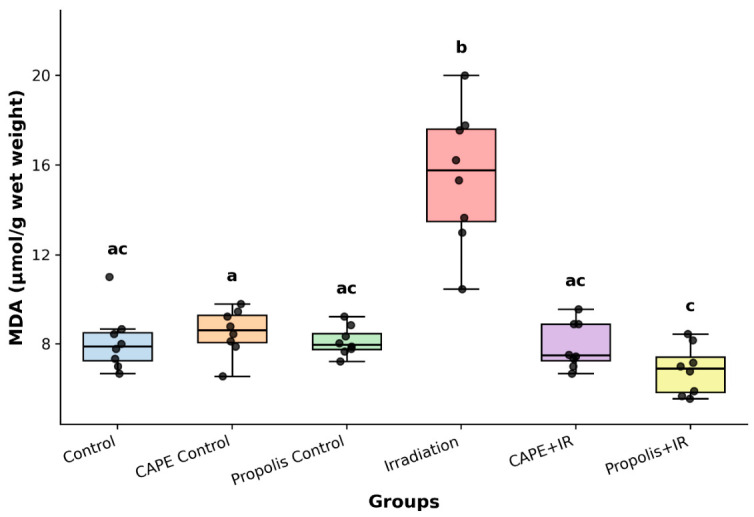
Mean ± SD of MDA level measured in the kidney of the rats (In box plots, if the letters on the boxes representing the groups are the same, there is no difference; if the letters are not the same, there is a difference between them). MDA levels were significantly increased in the Irradiation group compared with the other groups. CAPE and propolis treatment significantly reduced irradiation-induced MDA elevation.

Assessment of oxidative stress markers demonstrated a significant increase in XO activity in the IR group compared with all other experimental groups (*p* < 0.001). Similarly, MDA levels differed significantly among groups, reaching their highest values in the IR group, while markedly reduced levels were observed in the IR + propolis group (*p* < 0.001). These results indicate that ionizing radiation induces pronounced oxidative stress, whereas treatment with propolis and CAPE effectively attenuates this response, supporting their potential role as radioprotective agents (Figure 1 and Figure 2). In box plots, if the letters on the boxes representing the groups are the same, there is no difference; if the letters are not the same, there is a difference between them.

### 3.3. Nitrosative Stress Parameters

**Figure 3:**
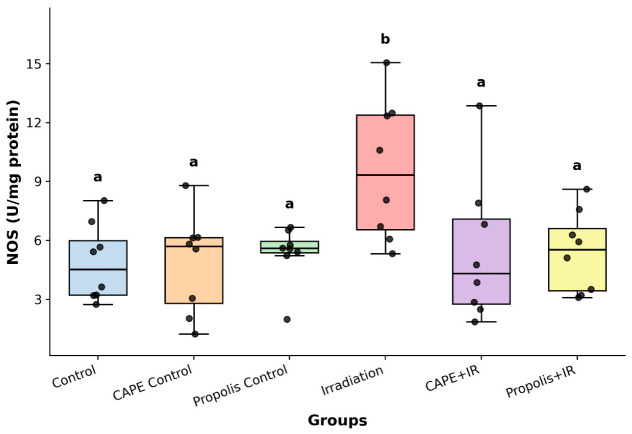
Mean ± SD of NOS activity measured in the kidney of the rats (In box plots, if the letters on the boxes representing the groups are the same, there is no difference; if the letters are not the same, there is a difference between them). NOS activity was significantly increased in the Irradiation group compared with all other groups. CAPE and propolis treatment significantly reduced irradiation-induced NOS activity, and the treated groups were not significantly different from the control groups.

**Figure 4:**
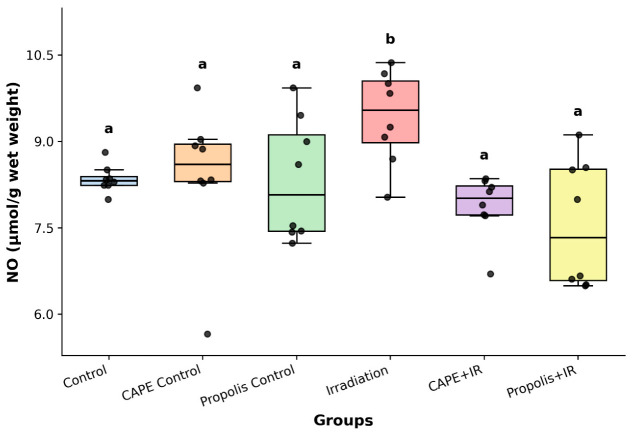
Mean ± SD of NO^•^ level measured in the kidney of the rats (In box plots, if the letters on the boxes representing the groups are the same, there is no difference; if the letters are not the same, there is a difference between them). NO levels were significantly increased in the Irradiation group compared with all other groups. CAPE and propolis treatment significantly reduced irradiation-induced NO levels, and the treated groups were not significantly different from the control groups.

Analysis of nitrosative stress parameters revealed that both NOS activity and NO^•^ levels were significantly elevated in the IR group relative to the other experimental groups (*p* = 0.007 and *p* < 0.005, respectively). Treatment with propolis and CAPE markedly reduced these increases, indicating their capacity to mitigate ionizing radiation–induced nitrosative stress. These findings further support the radioprotective potential of both compounds (Figure 3 and Figure 4). In box plots, if the letters on the boxes representing the groups are the same, there is no difference; if the letters are not the same, there is a difference between them.

### 3.4. Correlation Analysis

Correlation analyses were performed as exploratory analyses to evaluate possible relationships among antioxidant, oxidative, and nitrosative stress parameters within each experimental group. Given the relatively small sample size in each group (n = 8), the correlation results were interpreted cautiously, and *p*-values were adjusted for multiple comparisons using the Benjamini–Hochberg FDR method.

Several nominal associations were observed before multiple-comparison correction, including correlations between XO and MDA, SOD and GSH-Px, GSH-Px and NOS, SOD and GST, GSH-Px and XO, GSH-Px and MDA, GST and NOS, XO and NOS, and XO and MDA in different experimental groups. However, after FDR correction, these within-group correlations no longer reached statistical significance. Therefore, these findings should be considered exploratory and hypothesis-generating rather than confirmatory. Overall, the correlation patterns suggest possible interactions between antioxidant defense mechanisms and oxidative/nitrosative stress pathways, but these observations require confirmation in studies with larger sample sizes.

### 3.5. Heatmap and Hierarchical Clustering Analysis

**Figure 5:**
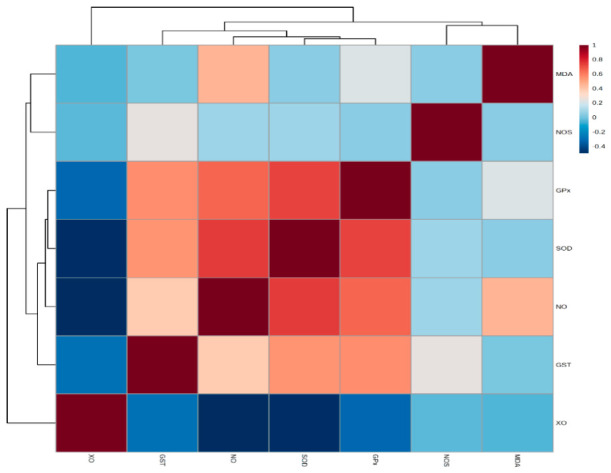
Heatmap of renal biochemical parameters.

**Figure 6:**
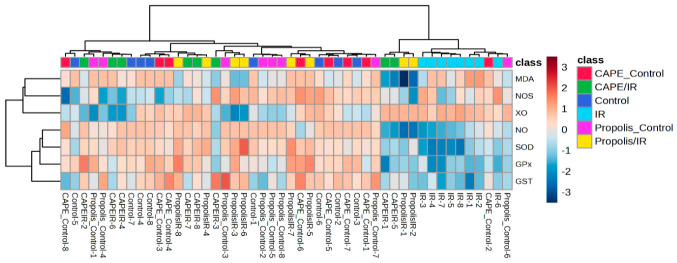
Hierarchical clustering of renal biochemical parameters.

Heatmap visualization combined with hierarchical clustering revealed distinct correlation patterns among the measured variables (Figure 5 and Figure 6). Examination of the variable-based dendrogram demonstrated that SOD, GSH-Px, and GST exhibited similar distribution profiles and clustered within the same branch, whereas XO was clearly segregated from this cluster. In contrast, MDA, NOS, and NO^•^ formed a separate cluster, indicating a distinct pattern relative to the other parameters. In the heatmap, red coloration represents relatively higher values, while blue indicates relatively lower values.

Sample-based clustering did not demonstrate complete separation between experimental groups; however, certain groups showed tendencies to cluster with similar samples, and partial overlaps between groups were observed. These findings suggest both shared and distinct biochemical response patterns across the experimental conditions.

### 3.6. Principal Component Analysis

**Figure 7:**
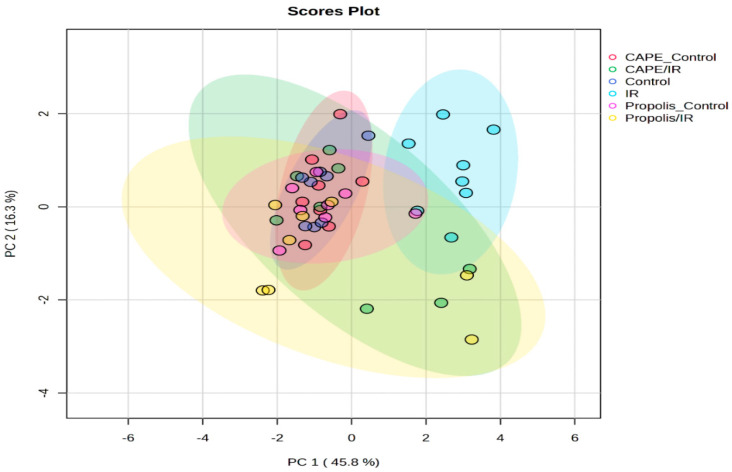
Principal component analysis (PCA) score plot of the study groups.

Principal component analysis (PCA) demonstrated a clear separation among the experimental groups (Figure 7). The first two principal components accounted for 62.1% of the total variance (PC1: 45.8%, PC2: 16.3%), indicating that a substantial proportion of the dataset variability could be captured within a two-dimensional space. The score plot revealed a distinct segregation of the IR group along the PC1 axis from the remaining groups. In contrast, control and treatment groups exhibited partial overlap, although they tended to cluster in different orientations.

The statistical significance of group-wise separation was confirmed permutational multivariate analysis of variance (PERMANOVA) (F = 6.5713; R^2^ = 0.43893; *p* = 0.001), indicating that approximately 44% of the total variance in the dataset could be attributed to group differences. These findings highlight a strong group-dependent effect on the overall biochemical profile.

### 3.7. PCA Biplot Analysis

**Figure 8:**
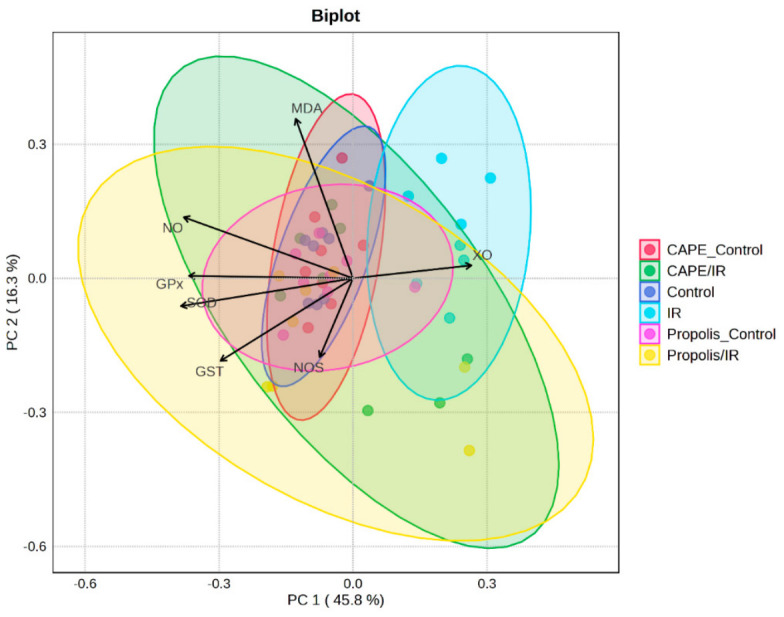
Principal component analysis (PCA) biplot of variable contributions.

Principal components analysis (PCA) biplot provided further insight into both the separation of experimental groups and the contribution of individual variables to this differentiation (Figure 8). XO exhibited a strong loading in the positive direction of PC1, indicating a major contribution to the variance along this axis. In contrast, SOD, GPx, and GST were positioned in the negative direction of PC1, suggesting an opposing pattern of association. MDA appeared to be more prominently distributed along the PC2 axis, while NOS and NO^•^ were oriented in distinct directions, reflecting variability and heterogeneity among these parameters. These patterns highlight the differential contributions of oxidative and nitrosative stress markers to the overall group discrimination.

### 3.8. PLS-DA Analysis

**Figure 9:**
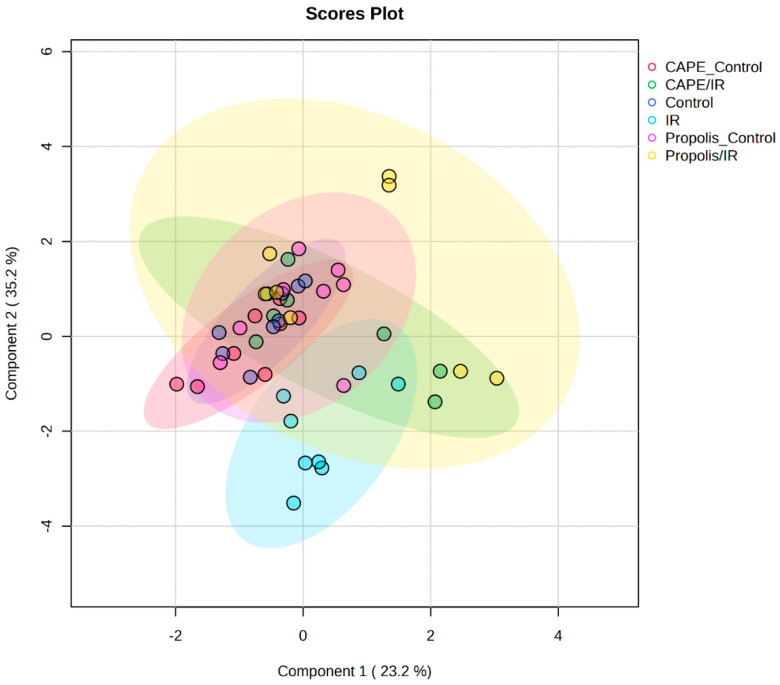
Partial least squares discriminant analysis (PLS-DA) score plot of the study groups.

Partial least squares discriminant analysis (PLS-DA) revealed a moderate degree of separation among the experimental groups in the score plot (Figure 9). The first two latent components accounted for a substantial proportion of the total variance, with the IR group distinctly localized in a region separate from the other groups. Examination of the score distribution indicated that control groups formed relatively compact clusters, whereas treatment groups exhibited greater dispersion, reflecting increased variability and partial overlap.

Notably, the IR + CAPE and IR + propolis groups occupied intermediate positions between the IR and control groups, without complete convergence with either. Evaluation of the 95% confidence ellipses confirmed the presence of partial group separation, although overlapping regions persisted. Overall, the PLS-DA model suggested a tendency toward group-dependent clustering within the dataset.

However, model validation indicated limited predictive performance. Cross-validation yielded relatively low accuracy values and negative Q^2^ scores, while permutation testing demonstrated that the model did not achieve statistical significance (*p* > 0.05). These findings suggest that, despite observable clustering trends, the discriminative capacity of the model may be insufficient for robust classification.

### 3.9. Variable Importance in Projection Analysis

**Figure 10:**
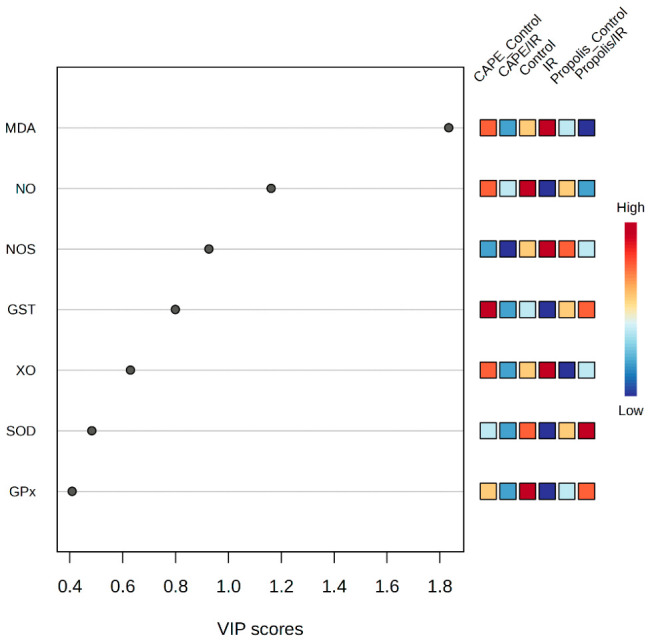
Variable importance in projection (VIP) scores of renal biochemical parameters(The black dots indicate the VIP score for each parameter. A VIP value greater than 1 indicates that the parameter significantly contributes to distinguishing groups.).

Variable importance in projection (VIP) scores derived from the PLS-DA model identified the variables contributing most prominently to group discrimination (Figure 10). Among the measured parameters, MDA exhibited the highest VIP score, indicating the strongest influence on class separation, followed by NO^•^ and NOS. In contrast, GSH-Px and SOD displayed comparatively lower VIP values, suggesting a more limited contribution to the model’s discriminative capacity. GST and XO were found to have moderate importance within the model. Overall, the distribution of VIP scores indicates that specific variables exert a disproportionately greater impact on group differentiation relative to others.

## 4. Discussion

The findings of the present study demonstrate that total cranial exposure to IR induces a significant increase in both oxidative and nitrosative stress in rat kidney tissue. Interestingly, these effects were not confined to directly irradiated tissues but were also evident at the systemic level. This observation is consistent with the concept of the RIBE, which has gained increasing attention in radiation biology. RIBE refers to the phenomenon whereby non-irradiated tissues exhibit biological responses, including oxidative stress, DNA damage, and inflammatory activation, as a consequence of signals originating from irradiated cells. The biochemical alterations observed in renal tissue in this study are therefore indicative of a bystander-mediated response [6,8,24].

In the present study, the activity of SOD, a key component of the antioxidant defense system, was markedly reduced in the IR group, whereas it was significantly elevated in the propolis- and CAPE-treated groups. This finding indicates that ionizing radiation suppresses antioxidant enzyme systems, thereby disrupting cellular redox homeostasis. Consistent with our results, previous studies have also reported that IR decreases SOD activity and weakens antioxidant defenses [2,8,25]. The observed increase in SOD activity following propolis and CAPE administration supports their roles as free radical scavengers and enhancers of antioxidant enzyme capacity. In contrast, the absence of significant changes in GSH-Px and GST activities suggests that these enzymes may represent more stable systems or exhibit delayed responsiveness under the experimental conditions.

Evaluation of oxidative stress parameters revealed that the significant increase in XO activity and MDA levels in the IR group reflects a marked elevation in lipid peroxidation and reactive oxygen species generation. The upregulation of XO contributes to the amplification of oxidative stress as a major source of superoxide production, whereas elevated MDA levels serve as a reliable indicator of cellular membrane damage [13,26,27]. These findings support the notion that ionizing radiation induces systemic oxidative injury. The observed reduction in these parameters following propolis and CAPE administration indicates that both compounds effectively suppress oxidative damage and limit lipid peroxidation. Of note, the markedly lower MDA levels in the propolis-treated group highlight the strong antioxidant capacity of this natural product.

From the perspective of nitrosative stress, the increases in NOS activity and NO^•^ levels observed in the IR group are particularly noteworthy. Excessive NO^•^ production can lead to the formation of peroxynitrite, resulting in protein nitration and subsequent cellular dysfunction. In the present study, the significant reductions in NOS activity and NO^•^ levels following propolis and CAPE administration indicate that these compounds exert modulatory effects not only on oxidative stress but also on nitrosative stress pathways [28,29]. CAPE, in particular, has been reported to suppress inflammatory responses and reduce NO production through inhibition of NF-κB signaling pathways [12].

Exploratory correlation analyses suggested possible associations among antioxidant defense, oxidative stress, and nitrosative stress parameters. However, given the small sample size within each group (n = 8) and the number of correlations tested, these findings should be interpreted with caution. After adjustment for multiple comparisons using the Benjamini–Hochberg FDR method, the within-group correlations did not retain statistical significance. Therefore, these correlation patterns should be considered hypothesis-generating rather than confirmatory. Although they may suggest potential interactions among antioxidant enzymes and oxidative/nitrosative stress markers, further studies with larger sample sizes are required to confirm these relationships.

Multivariate analyses enabled a comprehensive evaluation of the obtained findings. The clear separation of the IR group from the other groups in the PCA score plot indicates that ionizing radiation profoundly alters the biochemical profile. PERMANOVA results further confirmed that this separation was statistically significant. In the PCA biplot, the strong contribution of XO to group discrimination supports the central role of oxidative stress in this process. In contrast, the opposing positioning of SOD, GSH-Px, and GST suggests a counteractive response of the antioxidant defense systems.

PLS-DA indicated a tendency toward separation among the groups; however, the model exhibited limited classification performance. Negative Q^2^ values and non-significant permutation test results suggest poor predictive capability. This limitation may be attributable to the inherent complexity of biological systems and the relatively small sample size. Notably, VIP analysis identified MDA, NO^•^, and NOS as the most influential variables, underscoring the pivotal role of oxidative and nitrosative stress in driving group discrimination. Consistently, heatmap and hierarchical clustering analyses demonstrated that antioxidant enzymes and oxidative/nitrosative stress markers were segregated into distinct clusters. This pattern indicates that these parameters reflect different biological processes and that ionizing radiation affects these processes through divergent mechanisms.

## 5. Strengths and Limitations of the Study

A major strength of this study is that, as an animal experiment, it was not affected by confounding factors commonly encountered in human studies, such as comorbidities and interindividual variability. Furthermore, the direct use of kidney tissue to assess oxidative damage is an additional advantage. However, the study also has several limitations. It was designed as an animal experiment, and, by its nature, the parameters investigated were evaluated in a limited number of samples from a relatively small number of rats. Although statistical analyses were performed, further studies with larger sample sizes would be valuable to confirm and extend these findings. The results of the present study demonstrated that both Propolis and CAPE attenuated oxidative damage in kidney tissue by reducing oxidant parameters and enhancing antioxidant defenses. Nevertheless, further investigations are required to determine the optimal dosage and timing of Propolis and CAPE administration, as well as to evaluate their efficacy and potential clinical applicability in humans.

## 6. Conclusions

The present study demonstrates that total cranial ionizing radiation induces pronounced oxidative and nitrosative stress in kidney tissue located the direct irradiation field, suggesting that these effects may be attributed to the radiation-induced bystander effect. Administration of propolis and CAPE was shown to enhance antioxidant defense systems while significantly reducing oxidative and nitrosative stress markers. These findings support the potential of these natural compounds as radioprotective agents against radiation-induced systemic damage. However, further studies with larger sample sizes, extended follow-up periods, and comprehensive approaches incorporating molecular, histopathological, and functional analyses are required to validate these effects at the clinical level.

## Figures and Tables

**Table 1:** Mean ± SD values of antioxidative parameters measured in the kidney of the rats.

Groups	SOD (U/mg Protein)	GSH-Px (U/mg Protein)	GST (U/mg Protein)
Control	840.1 ± 72.1	303.4 ± 49.9	14.6 ± 2.8
CAPE Control	815.5 ± 78.5	310.5 ± 62.3	18.2 ± 5.4
Propolis Control	847.2 ± 80.4	271.4 ± 47.9	16.7 ± 6.6
IR	743.7 ± 80.2	303.3 ± 91.8	17.0 ± 4.8
CAPE + IR	914.9 ± 73.2 ^a^	286.7 ± 68.5	18.2 ± 8.5
Propolis + IR	1137.7 ± 195.6 ^a^	342.6 ± 68.8	17.8 ± 4.1

^a^: *p* < 0.0001 vs. irradiation group.

## Data Availability

Data generated or analyzed during this study are available from the corresponding author on reasonable request.
